# Comment on ‘Weight‐adjusted waist as an integrated index for fat, muscle and bone health in adults’

**DOI:** 10.1002/jcsm.13505

**Published:** 2024-06-05

**Authors:** Xiao Li

**Affiliations:** ^1^ First Clinical Medical College Shandong University of Traditional Chinese Medicine Jinan China

I read with interest the paper entitled ‘Weight‐adjusted Waist as an Integrated Index for Fat, Muscle and Bone Health in Adults’ by Kim et al.,^1^ which utilizes the KNHANES (Korean National Health and Nutrition Examination Survey) to innovatively investigate the association between a novel index for assessing obesity, the weight‐adjusted waist circumference index (WWI), and unhealthy body composition in the Korean population. WWI was found to be negatively correlated with bone and muscle mass but positively correlated with fat mass, and significantly higher outcomes of unhealthy body composition (high‐fat mass, low muscle mass and low bone mass) were found in higher quartiles of WWI than in lower quartiles (18.08 [95% confidence interval, CI, 4.32–75.61] for men and 6.36 [95% CI, 3.65–11.07] for women). Tissue dysfunction in muscle, bone and fat is closely related to human health and may be a risk factor for disease and death. Compared with traditional anthropometric measures for assessing obesity, WWI can better differentiate between fat and muscle mass and may help us better identify people at risk for unhealthy body composition. All in all, I think this is a very interesting study. But I also have some questions about the study.

Firstly, in the selection of covariates, the authors adjusted for important covariates such as age, smoking status and hypertension status in this study, which is excellent, but I noticed that the authors also adjusted for dyslipidaemia (including total cholesterol, triglycerides, LDL cholesterol and HDL cholesterol) during the course of the study, so may I ask the authors if they took into account the multiple covariances?

Secondly, can the authors complement the limitations of the remaining obesity indices in their study and thus explore whether WWI has a stronger association with unhealthy body composition than when using body mass index (BMI) or waist circumference (WC)? This is because the authors also mentioned in their study that WWI has unique advantages over the traditional assessment of obesity indices. And also, the authors plotted the receiver operating characteristic curve to analyse the predictive ability of WWI, so is it possible to consider comparing WWI with other obesity indices (e.g., WC, BMI, waist‐to‐height ratio, a body shape index [ABSI], etc.) to observe whether it is a better predictor of unhealthy body composition?

In conclusion, my suggestion is to make an already excellent study even better, and I also hope that the authors can focus on my questions so that readers will get more accurate conclusions from the study.

## Conflict of interest statement

The author declares that the research was conducted in the absence of any commercial or financial relationships that could be construed as a potential conflict of interest.

## Funding

The author declares that no financial support was received for the research, authorship and/or publication of this article.

## References

[1] Kim KJ , Son S , Kim KJ , Kim SG , Kim NH . Weight‐adjusted waist as an integrated index for fat, muscle and bone health in adults. J Cachexia Sarcopenia Muscle 2023;14:2196–2203. 37550773 10.1002/jcsm.13302 PMC10570086

